# Enhanced lion swarm optimization and elliptic curve cryptography scheme for secure cluster head selection and malware detection in IoT-WSN

**DOI:** 10.1038/s41598-024-81038-1

**Published:** 2024-12-02

**Authors:** Udaya Suriya Rajkumar D, Sathiyaraj R, Bharathi A, Mohan D, Vidyullatha Pellakuri

**Affiliations:** 1 Department of Computer Science and Engineering, Global Institute of Engineering and Technology, Melvisharam, Ranipet District, Tamil Nadu India; 2 Department of CSE, GITAM School of Technology, GITAM University, Bangalore, Karnataka India; 3 Department of Information Technology, Bannari Amman Institute of Technology, Sathyamangalam, Tamil Nadu India; 4 Department of Computer Science and Engineering, Global Institute of Engineering and Technology, Melvisharam, Ranipet District, Tamil Nadu India; 5 Department of Computer Science and Engineering, Koneru Lakshmaiah Education Foundation, Vaddeswaram, Guntur, Andhra Pradesh India

**Keywords:** Wireless sensor network, Internet of things, Attack detection, Enhanced lion swarm optimization algorithm and elliptic curve cryptography scheme, Computer science, Information technology

## Abstract

Wireless Sensor Networks present a significant issue for data routing because of the potential use of obtaining data from far locations with greater energy efficiency. Networks have become essential to modern concepts of the Internet of Things. The primary foundation for supporting diverse service-centric applications has continued to be the sensor node activity of both sensing phenomena in their local environs and relaying their results to centralized Base Stations. Malware detection and inadequate Cluster Heads node selection are issues with the current technology, resulting in a drastic decrease in the total Internet of Things-based performance of sensor networks. The paper proposes an Enhanced Lion Swarm Optimization (ELSO) and Elliptic Curve Cryptography (ECC) scheme for secure cluster head selection and malware detection in IoT-based Wireless Sensor Networks (WSNs). The paper includes network models, choice of Cluster Head (CH) and attack detection procedures. The proposed method chooses the Cluster Head with the best fitness function values, increasing data transmission speeds and energy efficiencies. Minimum Hop Detection has been implemented to provide the best routing paths against attack nodes. Security level for quick data transmissions via the Internet of Things using Wireless Sensor Networks strengthen sinkhole attacks and black hole nodes, which are successfully removed using this method. The proposed method integrates the use of Lion Swarm Optimization and Elliptic Curve Cryptography (ECC) enhances network security by ensuring secure data transmission and preventing unauthorized access, which is particularly important in IoT-WSN environments. The proposed method achieves less End delay, increased throughput of 93%, lower energy utilization of 4%, increased network lifetime of up to 96%, Packet Delivery Ratio of up to 98% and 97% of malicious node detection efficiently compared to existing methods.

## Introduction

In Wireless sensor networks, dispersed independent sensors are utilized to monitor ambient and atmospheric conditions and to collectively transfer their data from the end-to-end network to the central location. In essence, the WSN is utilized to gather sensed data from far off, inaccessible regions based on the necessary direction. In many applications, the data is sensed by wireless sensors that are powered by batteries to get around this issue. The WSN with battery- powered applications does, however, deal with a number of issues, such as limited lifespan and excessive energy consumption. Outdoor short range sensor networks then replace the battery powered wireless sensors. These short-range sensor networks include fixed nodes at ground level, so sequential changes only occur when an object travels close to the sensor. In outdoor wireless broadband systems, the transceivers are placed close to the ground to ensure improved propagation. In essence, one of the most difficult tasks in a WSN is the interchange of data between sensor nodes^1^.

Their adaptability is due to their composition of tiny, low-cost sensor nodes that can sense their environment^2^. These nodes are scattered around a region for collection of data and are frequently extremely small. A stronger BSs (Base Stations) or sink node is present them with, and its job is to receive and analyse the data detected by the other nodes. These nodes do, however, have design restrictions, such as little battery life, tiny memory, processing power and constrained computational. As an outcome, many application requirement designs, such security and threat detection, are challenged by these limits. IoT has become a useful, adaptable, and interoperable network of electronics. Developments in networks, communications and computer technologies, have resulted in the growth of the Internet of things out of its beginning and are now recognized as the subsequent ground-breaking innovation in turning the Internet medium into a cohesive Internet^3^. IoT makes use of WSNs in the collection, share, and dispatch of remote for full utilization of its potential in useful applications and services. However, many and major security assaults may pose a danger to data delivery through the Internet.

The closest nodes to the ideal site are designated as CHs during selection in the majority of current research utilizing clustering for WSNs. As a result, clustering mostly places CHs near the cluster centre, and operation problems may occur when the recommended CH placements and actual node positions diverge^4^. To start with, the network lifespan is shortened and energy consumption increases while identifying the closest nodes after establishing CHs. Second, a node from another cluster could be incorrectly employed as CHs due to the huge divergence between the ideal Locations of CHs and real Locations of CHs. The proximity to optimum positions of clusters is considered while selecting CHs for many clusters resulting in fewer clusters than CHs or less-than-ideal performances. This implies clustering methods should consider characteristics of WSNs like true node placements.

Security requirement is provided as one of the core needs for all sorts of WSN applications during routing. However, the inhospitable conditions in which WSNs are often placed leave them open to many security threats. Additionally, all nodes transmit their data to BSs in WSNs utilize many-to-one communication patterns resulting in extra liabilities^5^. This makes, WSNs susceptible to the collection of inside and outside attacks. Attacks from outside the network, which try to tamper with network operation, are known as outsider attacks. An attacker that gains access to sensor nodes can launch attacks against domains or trip off other assaults. This is known as an insider attack. Sinkhole or black hole insider attacks are characterized as active route disruption attacks on network layers^6^. In these types of attack, the attacker nodes pull other nodes towards themselves by advertising themselves as high-quality routing paths to BSs or they seem closer to BSs than other nodes. As a result, nodes frequently use malicious node routes, which can change, spoof, or delete delivered packets and prevent BSs from receiving correct or complete information. The ability of sinkhole to facilitate additional attacks like worm hole attacks and selective forwarding attacks makes them one of the most harmful attacks on WSNs.

The adaptive management strategies and dynamic search space limitations that ELSO uses make it better than traditional Lion Optimization Algorithms (LOA) at avoiding local optima and converging to global solutions. This improves the algorithm’s exploration and exploitation capabilities. This makes it an ideal choice for settings with limited resources since it improves energy economy, network duration, and latency reduction performance. In addition, the research makes ELSO particularly suitable for IoT networks by modifying the optimization procedure to handle performance parameters like energy consumption and network scalability, which are essential for large-scale installations.

- The primary contribution and novelty of the paper is detecting attacks on IoT-based WSNs. Several studies and approaches have been developed previously. However, malware detection has not seen considerable improvement.
- The present methods have issues with attack detection in the choice of WSNs and optimum CH. The ELSO-ECC strategy suggested in this article helps enhance the overall performance of WSNs while overcoming the abovementioned concerns.
- The key contributions of this research are network modelling; CH decision-making made using the ELSO algorithm, and attack detections during routing processes. The recommended method can produce improved outcomes for IoT-based WSNs regarding network longevity, decreased energy usage, increased throughput, minimum packet loss ratio and malicious node detection.
- This research proposes an ELSO approach and the ECC scheme to solve these problems. The suggested ELSO enhances data transmission speeds and energy efficiency by selecting CHs with the best fitness function values.
- Then, minimum hop detection is used to determine the optimum routing pathways against attack nodes. Stronger sinkhole attacks and black hole nodes that are effectively eliminated using the ECC technique, Security level for rapid data transfer via IoT using WSNs.

The rest of the paper is structured as follows: In Sect. 2, a quick summary of the research on CHs, attack detection, and security measures in WSNs is provided. Section 3 provides specifics on the suggested technique for the ELSO with ECC method. Section 4 deals with the performance analysis. Section 5 the findings.

## Related work

A robust Federated Learning architecture of Android malware applications for IIoT was presented in^8^ under the name Fed-IIoT. The Fed-IIoT contains two components (i) Two dynamic poisoning attacks methods on generative adversarial networks and Federated Generative Adversarial Networks that activate data on the participant side with the removal of anomalies seen in A3GANS and modification of two GAN based algorithms on the server side and ii) on the server side, attempts to tracking the overall model and construction of a strong collaboration training method through removal of anomalies in A3GANs (aggregate by GANs) and changes in two GAN-based countermeasure methods, seeks to monitor the global model and build a robust cooperation training model. One of Fed-key IIoT’s benefits is that devices may effectively connect with one another and safely participate in the IIoT without worrying about their privacy. The study includes solutions using three IoT datasets and a variety of attributes. The research reveals A3GANs defensive strategy as 8% more accurate than existing technologies with robust ensuring of data privacy for Android mobile users, also with support attack and defence algorithm with improved accuracy rates.

In^7^ presented a unique IDASA (intrusion detection algorithm) offers protection against sinkhole attacks based on neighbour information. In contrast to conventional intrusion detection techniques, IDASA fully utilizes sensor node neighbour information in findings sinkhole nodes. Together with assessment of IDASA in MATLAB in terms of the reliability of malicious node identification, loss of packet rate, usage of energy, and performance of the network. Simulation findings demonstrate that IDASA executes improved than other comparable algorithms. In^10^, the cluster head nodes need for routing are selected utilizing a harmony search algorithm, while the nodes in the middle are chosen using a black widow optimization strategy.

In^11^, researchers identified IoT applications for energy harvesting in WSNs. This study suggested Trust-Based Secure routing technique for enhancing security and gathering as much energy as feasible for sensor networks. Their method used different channels for sources and sink communications, allowing data to be independently validated for security. Additionally, their trace back strategy identified rogue nodes using probability-based strategies which handled problems of security and battery levels. FLION (Fractional Lion) clustering method, a capable optimization-based approach for building an energy-efficient routing path. Their study used clustering approach to extend energy and lifespan of network nodes with selections of CHs^12^. Moreover, using five objectives intra cluster, inter cluster, CHs normal nodes, and delay distances their FLION multi objective clustering technique developed novel fitness functions. Fast cluster centroid was discovered by the fitness functions for efficient routing paths. Several clustering methods, including LEACH and PSO (Particle Swarm Optimization) and ABC (Artificial Bee Colony) clustering algorithm were compared with the study’s proposed approach. Their findings demonstrated FLION clustering maximized lifetime of WSNs.

In^13^ presented a novel LSO method based on multi-agent frameworks. The scheme worked on increasing lion swarm optimization’s global exploring capabilities and blended LSO with multi-agent systems to improve efficacy and accuracy. Search strategies were iterative processes based on information from group and their surroundings. The study’s scheme substantially improved performances in terms of efficiencies and robustness while dealing with power system’s economic load distributions. Their experimental findings on power systems demonstrated the efficacy of this novel method. ECC is used for safe key distribution and data transfer. ECC uses smaller keys but yet offers the same level of protection. Additionally, this system includes mutual authentication between the base station server and sink node. For two-way authentication, it employs the timestamp value, registered random number, and sinks ID hash value. Additionally, the ECC approach recognizes replay attacks using the timestamp value. Overall, it demonstrates a more effective security solution for WSNs for medical equipment^14^.

In^22^ suggested the Modified Salp Swarm Algorithm (SSA) for the Best Fitness Function for the increasing WSN Lifetime. This work presents a modified SSA to solve the energy-efficient clustering and routing issue in WSNs based on the d-kp formulation. The author examines the difficulty of this problem and provides the findings. A metaheuristic algorithm, SSA takes information from how salps swim in the water. Additionally, the author shows that maximizing a long-term metric like FND (time of first node death), a popular measure for network longevity, using a polynomial time fitness function is impossible. These results suggest that more effective algorithms are needed to tackle this complicated challenge, affecting how energy-efficient WSNs are designed.

In^23^ proposed the Graph Neural Networks with coverage metrics for Energy-efficient clustering for dense WSN. This strategy aims to provide an energy-efficient WSN clustering protocol by balancing the nodes’ energy usage. The author offers a distributed cluster head selection approach that uses every cluster individually. With multi-hop routing, WSNs may avoid energy-intensive long-distance communication, another critical energy efficiency component. According to the suggested centralized routing strategy, the clusters will have dedicated paths to the main hub. As a result, the hotspot issue is avoided, and the energy consumption of relay nodes is uniform. The suggested strategy was tested in simulations alongside several state-of-the-art alternatives to ensure its efficacy. The method outperforms competing protocols in the real world based on lifespan and coverage parameters.

In^24^ recommended the type-3 fuzzy system in WSN for an efficient grid-based clustering algorithm. Primarily, the author discussed and divided uncertainty into two groups: main uncertainties and secondary uncertainties. Second, the author has used a Type-3 fuzzy system to deal with further uncertainty. Secondly, the author has generated unequal clusters and balanced the load according to the position of the base station using an adaptable imaginary grid. In the fourth place, new adaptive imaginary grid updates have allowed for operational centralized and decentralized clustering. As a last step, the author has proportionately calculated the threshold level for each cluster by looking at the energy of nodes in that cluster. These improved results show that the network will last longer than similar solutions.

In^25^ introduced the Weighted RNN and Optimal Path Selection for Detection and Mitigation of Vampire Attacks with Secure Routing in WSN. In the beginning, all of the sensor nodes in the WSN system have their data properties gathered. In addition, an Enhanced Golf Optimization Algorithm (EGOA) is used to improve the weight values in a Weighted Recurrent Neural Network (WRNN), which is then used to identify vampire attacks. Various node attributes, including the number of broadcasts, energy, and Packet Received Ratio (PRR), allow for the effective separation of the discovered vampire nodes. Removing the vampire nodes from the network may begin the attack mitigation procedure, and the surviving nodes can be used for routing.

In^26^ presented the Cluster-Based Secure Optimal Routing for Secure and Energy-Aware Data Transmission for IoT-WSNs. The best Cluster Heads (CHs) are chosen for Data Aggregation (DA) using the Boltzmann Selection Probability-centric Gravitational Search Algorithm (BSP-GSA). These tasks are executed after the network’s nodes have been initialized. Then, the non-cluster members are joined with a neighbouring CH to create a cluster. In the same way as non-cluster members of the CHs collect data, they also encrypt it using the Improved Elliptical Curve Cryptography (IECC) process, which ensures the data’s security. Afterwards, the encrypted data is sent to the Base Station (BS) via the best path chosen using a Deep Learning (DL) algorithm (MUMLP) that takes into account a new fitness function.

In^27^ discussed the exponential polynomial kernel-centered deep neural networks (EPK-DNN) in WSN for an accurate attack detection framework. The first stage of the EPK-DNN method is training the attack detection system. The first step is to preprocess the input data. Then, it may use the pre-processed data to extract attributes during training. Step two involves selecting the primary features using the linear scaling-based BAT optimization (LS-BAT). Once the characteristics are selected, the EPK-DNN is trained to identify WSN assaults. Step three involves initializing the WSN network and clustering the sensor nodes using the Damerau-Levenshtein-based K-means algorithm (DL-K-Means). Cluster heads are chosen to collect sensor data using the swap, displacement, and reversion-centred rock hyraxes swarm optimization technique.

In^28^ deliberated the Adaptive Neuro-Fuzzy Inference System (ANFIS) classifier for Cluster-Based Malicious Node Detection System for Mobile Ad-Hoc Networks. The ANFIS classifier qualifies the conviction parameters to be extracted from trustworthy and malicious nodes. Furthermore, in the testing mode of the classifier, the individual MANET nodes are categorized. As the number of hostile nodes in the network grows, its performance will deteriorate. A rogue node entering a network may disrupt throughput, packet delivery ratio, energy consumption, detection rate, kink failures and accuracy value. In^29^ investigated the hybrid machine learning models for Secure localization methods in wireless sensor networks against routing attacks. This study suggests a method for secure localization and routing threat detection in wireless sensor networks using hybrid optimized machine learning techniques for optimum distance, location, and data transfer. These methods aim to determine the ideal placement and spacing between sensors. In^30^ proposed the Philippine Eagle (MKMPE) optimization and updated k-means-based cluster head selection for safe MANET routing. To guarantee that data packets reach their destination node without any loss, the modified k-means technique (MKM) is first used to select the best cluster head (CH) from the cluster group.

In^31^ proposed the Fuzzy-Based IDS routing technique in WSNs for Effective data transmission through energy-efficient clustering. At the outset, data collection began with the nodes being started and distributed randomly across the network. Using a round-robin management technique, LEACH selects cluster heads (CHs) at random and distributes them across the nodes to guarantee equitable energy dissipation. The intrusion-detection process was then used to find out whether there were any intruders on the network. One way that the WSN dealt with rogue nodes was by using a Fuzzy interference rule. After that, an ANN was used to separate the malicious nodes from the suspicious ones. The Intelligent Clustering Routing Approach (ICRA) for UAV Ad Hoc Networks was proposed in^32^. The three primary components of the ICRA are routing, clustering, and clustering strategy modification. Each cluster node has to assess its own utility. In order to maintain high topological stability and a long network lifetime in various network states, the adjustment module for the reinforcement learning-based clustering approach must constantly learn the advantages of implementing various strategies to determine the usefulness of nodes in a particular network state. The clustering strategy adjustment module may evaluate the network’s state and select the most effective clustering technique by applying what it has learned.

In^33^ proposed the Privacy-Preserving Reputation Updating Scheme (PPRU) for Cloud-Assisted Vehicular Networks. Using the Paillier algorithms and Elliptic Curve Cryptography (ECC), the author of this paper proposes a new scheme for cloud-assisted vehicular networks called Privacy-Preserving Reputation Updating (PPRU). This scheme allows the honest-but-curious Cloud Service Provider (CSP) to collect and preprocess reputation feedback in a privacy-preserving manner, reducing computation overheads on the TA side by about 81.36% and communication overheads by 83.88% (Table 1).

**Table 1:** Summary of the related works.

References	Methods used	Advantages	Limitations
^8^	Federated Generative Adversarial Networks (A3GANS) and two GAN-based algorithms	Effective privacy protection, improved accuracy by 8%, robust data privacy for Android users	Complexity in GAN-based algorithms and federated learning
^9^	Delay Conscious routing protocol	Effectively used to Multi point relay nodes	Several Problems including limited density, high speed nodes and abrupt dynamic topology.
^7^	Intrusion detection algorithm based on neighbour information	Better identification of sinkhole nodes, improved network performance, energy-efficient	Dependence on neighbour information, might not scale well in larger networks
^10^	Human-interactive, visual-based anomaly detection devices	Real-time wormhole attack detection, visual representation of security risks	Limited to IP-enabled WSNs, visual tool scalability concerns
^11^	Trust-based routing technique, FLION clustering	Enhanced security, energy-efficient routing, extended network lifespan	Potential overhead from multiple trust evaluations, complexity in implementation
^13^	Lion Swarm Optimization (LSO) with multi-agent frameworks	Improved global exploration capabilities, enhanced accuracy and efficiency	Increased computational overhead, complexity in multi-agent system integration
^19^	Swarm Intelligence-based Clustering Technique	Improve network throughput	Unbalanced power usage is a common concern in cluster based routing
^22^	Modified SSA for clustering and routing, polynomial time fitness function	Maximized network lifespan, efficient energy use	Complexity in fitness function formulation, potential overfitting
^23^	Distributed cluster head selection, multi-hop routing	Balanced energy consumption, avoided hotspot issues, effective for dense WSNs	Complexity in GNN implementation, potential scalability issues
^24^	Grid-based clustering, Type-3 fuzzy system	Enhanced network longevity, adaptable clustering	Complexity in fuzzy system, potential issues with real-time adaptability
^25^	Weighted Recurrent Neural Network, Enhanced Golf Optimization Algorithm (EGOA)	Effective vampire attack detection, optimized routing paths	Complexity in RNN and optimization algorithm, computational overhead
^26^	Boltzmann Selection Probability-centric Gravitational Search Algorithm (BSP-GSA), IECC, MUMLP	Secure data transmission, energy-aware routing, cloud storage integration	Complexity in multiple algorithm integration, potential latency in cloud storage
^27^	EPK-DNN, LS-BAT, DL-K-Means	High detection accuracy, robust against various attacks	High computational requirements, complexity in multi-algorithm integration
^28^	Adaptive Neuro-Fuzzy Inference System (ANFIS), MATLAB integration	Effective malicious node detection, adaptable classification	Performance degradation with increasing hostile nodes, complexity in MATLAB integration
^29^	K-means clustering, hybrid optimized machine learning techniques	High localization accuracy, effective routing threat detection	Dependence on specific datasets, complexity in hybrid model implementation
^30^	Modified k-means, Philippine Eagle (PE) optimization	Secure and efficient routing, improved detection rate	Potential issues with scalability, computational complexity
^31^	LEACH, Fuzzy interference rule, ANN	High specificity, accuracy, and sensitivity, effective data transmission	Potential energy consumption from ANN, complexity in the integration of multiple methods
^32^	Blockchain based identification Strategy	It is used to improve the level of compatibility among blockchain and IoT	End-to-end security is challenging due to the intricacy of IoT communication and different resource capacities.

Energy efficiency, scalability, security vulnerabilities, and optimization complexity are the main focal points of the current security and clustering issues in IoT-WSNs. While traditional approaches such as LEACH do not waste energy, they are not secure enough and cannot adjust to changing network circumstances. PSO-based approaches enhance clustering performance, although computational cost may be introduced, particularly in large networks. To remedy these problems, the ELSO-ECC method proposes several innovative improvements. A more secure and efficient method for selecting cluster heads is provided by combining Lion Swarm Optimization (LSO) with Elliptic Curve Cryptography (ECC), which improves the system’s flexibility to changing network circumstances. Addressing the security weaknesses of traditional approaches, ECC guarantees solid data security with minimum computing cost, making it perfect for resource-constrained IoT devices. The method also maintains performance as the network expands, balancing optimization with security. It works well in huge networks.

## Proposed methodology

This article proposes the ELSO + ECC algorithm for effective malware detection over Io T-based WSNs. The system concept, CH choices using ELSO, and attack detection using the ECC algorithm comprise the primary contribution of this article. To improve network lifespan, energy efficiency, and security in IoT-based WSNs, the study shows that the ECC scheme and the improved ELSO algorithm work together to provide a complete solution. Using this method, one may improve data security against typical network assaults and optimize cluster head selection to make the network last longer and run more efficiently. The suggested method’s efficacy in building a robust and efficient IoT network is shown by its ability to effectively handle sinkhole and black hole attacks and improve data transmission rates. Elliptic Curve Cryptography (ECC) and Malware Detection have been integrated to ensure the security and dependability of IoT networks. Encryption Key Cryptography (ECC) protects data transmissions and communications between nodes, stops prying eyes from seeing through sensitive data, and ensures it stays intact. Due to the significant susceptibility of IoT devices to malware and illegal access, it is crucial to combine ECC with MHD to safeguard data and identify possible malware risks during transmission. This method enhances the network’s security and stability, making it an ideal choice for IoT settings with limited resources. Our technique’s cluster head (CH) selection uses the Ant Lion Optimization (ALO) algorithm. An optimization method called ALO takes its cues from how ant lion’s hunt. Because of the mobility of nodes and the potential for topology changes in IoT-WSNs, it excels at optimization tasks in complex and dynamic contexts. To ensure effective data transmission and appropriate resource allocation, ALO assists in choosing the best cluster heads, considering several aspects such as energy levels, distance, and network stability. With this revised publication, we want to provide a more thorough explanation of ALO’s function, especially concerning the selection of cluster heads.

### System model

**Fig. 1:**
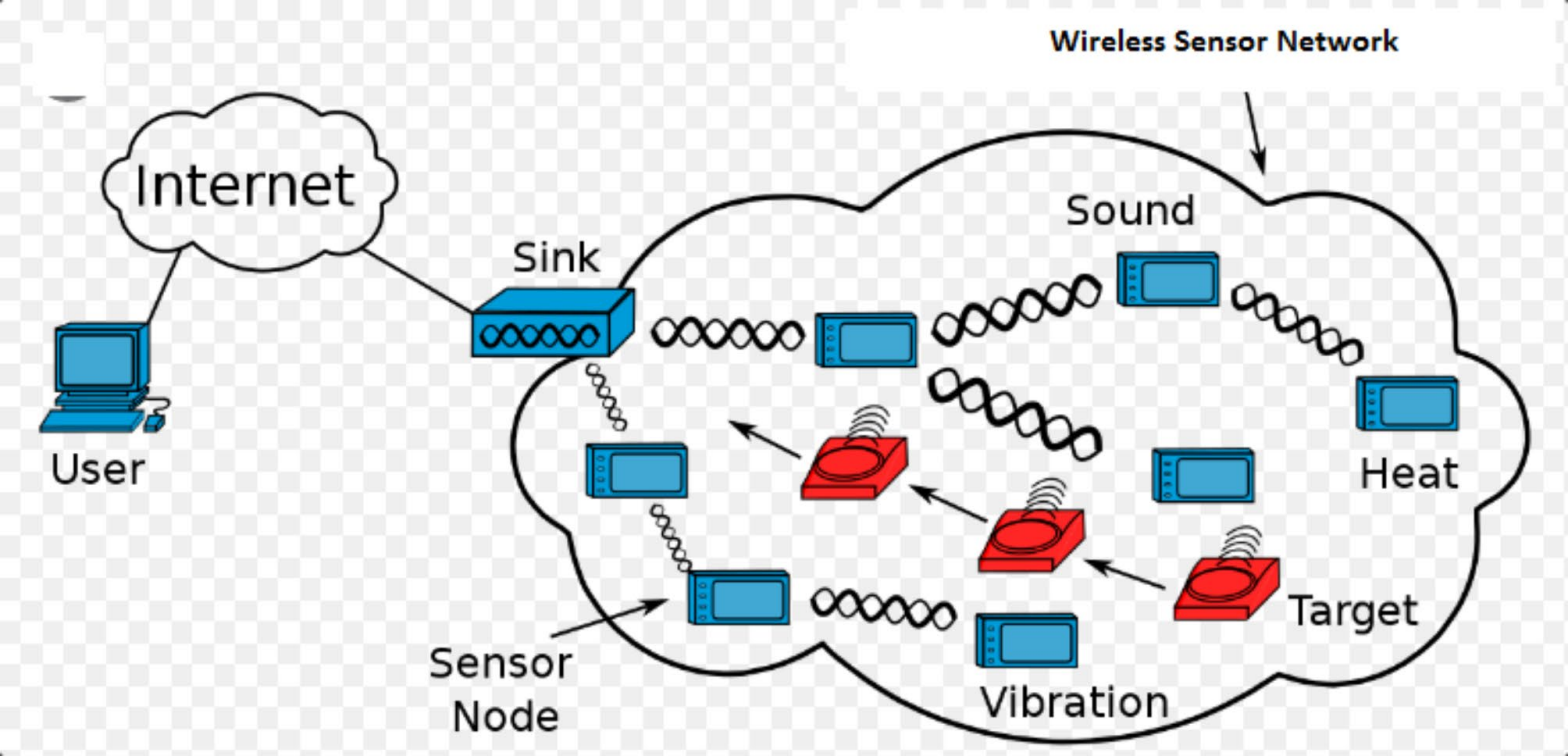
Structure of WSNs.

Typically, sensors are placed in the environment for sensing things and sending their findings to a centralized BS. The system model has assumed the operation of IoT sensors inside clusters to have the ability to interact with one another. The cluster count and sensor density of the IoT sensor nature are both influenced by a variety of different factors. The failure of a single sensor is tolerated more when there are several clusters present in the network than if clusters were absent. Intra-cluster sensor nodes need location close to one another for ensuring the active connection of peer sensors of a specific cluster connecting their separate sensing for validation. Additionally, each network cluster will have preconfigured CHs that will do the connection with the centralized BSs and collection of data inside the cluster. The impact of extensive communication between the sensor nodes and BSs, helps them avoiding quick battery fatigue through use of by using a hierarchy of IoT sensors^15^. Despite the necessity for the IoT sensor network to function, CHs carrying greater compute, communication, and energy resources than the cluster’s ordinary sensors are recommended. The construction of WSNs is shown in Fig. 1.

The IoT network is a standalone network, making it simple for an attacker to be arrogant about routing efficiency. IoT keeps sensors connected in an autonomous, secure manner. Protecting network operations from hostile nodes like sinkhole and black hole nodes is the major challenge in network security. When a person is engaged in harmful behaviour, it is indication for frequent use of network resources for their job but with no desire share to resources with other peers. In a network, the nodes themselves handle all routing (route construction and management). The routing information can be destroyed by hostile nodes using pronounced lengthy, ineffective, or fictitious routes, or by removing network packets rather than forwarding them, to disrupt network functioning. A “sinkhole” is created in a sinkhole attack when an adversarial node acting as an appealing node causes other nodes to share data. An attacker can take over a network’s routing protocols and make themselves the go-to for all data flow. The outcome is that the sinkhole node receives data from the majority or all of the inputs to the network. After that, a malicious node may change, delete, or eavesdrop on the information. Not only can this result in lost data, but it may also slow down the network and deplete the power of nearby nodes quicker as they route their data via the sinkhole. An illegal node known as a black hole node would pretend to be a route to the destination before draining all incoming data and never sending it on. It causes data loss and communication failures by creating a “black hole” into which data disappears. Potentially leading other nodes to reroute their traffic via the infected black hole node and causing crucial data loss, the node might cause major interruptions in network operations.

### Formations of clusters

Clustering is an effective approach used in WSNs for preservation of the sensor nodes irreplaceable battery power. The KMC (K-Means Clustering) technique is largely based on Euclidean distances, with the influence of residual energies of the nodes on the cluster head selection. As a result, in this instance, the central node collects information on each nodes position, amount of remaining energy, and node id stores in a list. After gathering this data from each node, the KMC method is used for a beginning clustering. It generates a specified number of clusters from the data and attempts to minimize an objective function.

1 $$J=\sum_{j=1}^{k}\sum_{i=1}^{x}\left|\right|{x}_{i}^{\left(j\right)}-{c}_{j}{\left|\right|}^{2}$$

Where$\left|\right|{x}_{i}^{\left(j\right)}-{c}_{j}{\left|\right|}^{2}$are the selected distance measures between data points${x}_{i}^{\left(j\right)}$ and cluster centres and where ${c}_{j}$ indicates distances the of n data points from their respective cluster centres’. It presumes the dispersal of the random disposal of sensor to with ability of communicate the sink with their locations. Sink may use the KMC technique for generation of the necessary number of clusters based on the numerous of network nodes after it has all of the positions of nodes. Then, all network sensors receive CHs list^16^. The CH designated sensor nodes then begin acting as CHs and broadcasting their existence across the network.

Use of this method, helps CHs with the need of less energy to convey data. For data transfer, the communication range between CHs and sensor nodes is kept to a minimum. The sensor node selects a different node when the distance between CHs and it exceeds the threshold as CHs are based on Euclidean distances. Nodes with good residual energy and improved channel circumstances are considered for the enhancement of the life span of the WSN’s. Following the creation of clusters and the clusters completion of CHs, the system computes the route based on CHs’ placements. The entire data set is collected and sent to the base station for processing. It spends less time and moves farther when visiting every cluster to collect data from CHs in order to preserve battery life. It is also necessary to take into account the speed and the distance among the clusters^21^.

### Selections of CHs using ELSO algorithm

The ELSOA technique is suggested in this work to choose the best CHs for enhancing the overall network performance more quickly. One of the most prevalent problems with routing protocols that must be considered is the choice of CHs. A subset of each source node’s neighbour is chosen. Multiple factors including connection qualities, physical distances, and residual energies were used to choose these nodes. To avoid collisions and redundant packet transmissions, nodes-maintained data packets for certain lengths of time determined by variety of criteria like distances, speed of sound propagation, transmission ranges, and residual energies. The best nodes had the least holding time, which maximized possibilities of data packets being properly passed.

The measures used to choose the future CHs have a direct impact on the overall efficacy of routing procedures. A residual energy metre is used to balance the energy between nodes. Link quality is another important component that has a direct impact on enhancing the packet delivery ratio and minimizing energy consumption. The use of a depth measure saves energy since each node estimates its own depth, whereas the sink may calculate the real distance using signals from beacons. Therefore, it is crucial to create a CH selection algorithm based on many parameters that chooses trustworthy and energy-efficient CHs in order to lower energy consumption, lower network traffic, and guarantee data delivery.

This study, ELSOA is presented for the best CH selection inside the provided network. A stochastic optimization tool is the LSOA meta heuristic algorithm. Each iteration of a meta heuristic algorithm might produce a distinct solution to the issue. Because of its unique social behaviour, the lion is the world’s strongest mammal. Lions live in groups known as prides, and both resident males and females assist in births. Territory refers to the area in which pride lives. The nomadic lion requires lions and cubs to defend it from assaults by rival prides^17^. If the territorial lion is beaten by the nomadic lion, the territorial lion will murder or drive away the territorial lion, and to establish its territoriality, it resolves also kill the lost lion’s pups before compelling the female lion to go into estrus and mate in order to generate offspring. When the territory cub reaches sexual maturity, if it is stronger than the territorial lion, it may kill it to seize control of the pride. The new, more powerful lion kills the territorial sluggish lion’s young and gets ready to have its own cubs. Ensuring energy efficiency is paramount for IoT devices since they often function in areas with limited resources. By factoring in energy use, the fitness function guarantees that the chosen cluster heads and routing pathways minimize energy usage, extending the network’s lifespan. The distance parameter is a key component in minimizing communication delays and guaranteeing the shortest route for data transfer. By considering physical distance, the algorithm finds the optimal path for data transmission, decreasing the total network latency.

Lions may transition between their two social organization kinds, from residents to nomads and vice versa. Lions have two different social behaviour patterns: residents and nomads. Based on the actions of two lions, LOA searches for the best solutions.

**Fig. 2:**
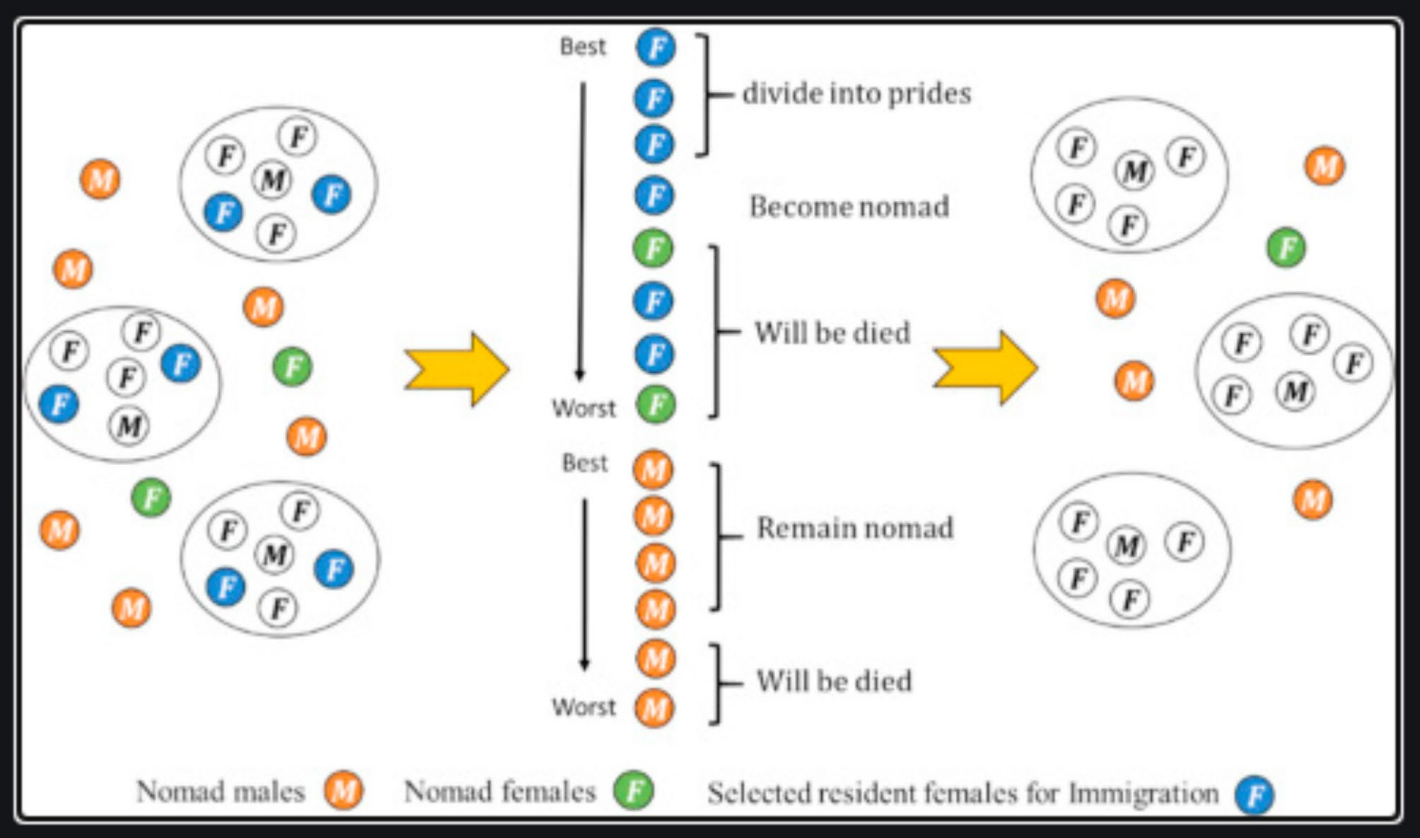
Depiction of LSO algorithm.

- The resident males (lions and cubs) and migratory males engage in territorial defence. According to this behaviour, the algorithm compares the novel solutions (nomadic lion) with the previous solution (territorial lion), and if the new solution is superior, the previous old solutions are destroyed.
- Prior territorial males and new territorial males compete for territories. By working in this manner, the algorithm will only store the best male and female solutions over fresh responses and delete any other solutions from the pride, preserving the improved solutions while eliminating the undesired ones (selection operation). The LSO method is depicted in Fig. 2.

One novel aspect of the research is its emphasis on how optimal clustering and ECC might reduce the impact of sinkhole and black hole attacks. Although other methods tackle these assaults separately, the study presents an integrated strategy that offers a whole answer. It combines stronger data encryption with superior cluster head selection, making it more resistant to these prevalent dangers. Ensuring that security advancements do not dramatically increase energy consumption is a typical concern in WSNs. To address this, ELSO’s energy-efficient clustering tactics are combined with the innovative use of ECC, which is recognized for its strong security with relatively minimal computing overhead.

The detection system has been optimized to be adaptable to new and developing malware threats by using an anomaly-based method to spot strange patterns in network activity. Elliptic Curve Cryptography (ECC) is included in the system to improve security. It protects feature data while it is being sent and guarantees that critical information remains concealed. This two-pronged strategy is ideal for low-resource Internet of Things (IoT) and wireless sensor network (WSN) nodes because it combines strong encryption with effective optimization. Deterministic performance measurements verify the suggested strategy and document encouraging outcomes, including computational delay, false positive rate, and detection accuracy. Unfortunately, there are several information gaps in the research, such as the training and testing datasets, the feature extraction preprocessing methods, and the real-world deployment circumstances.

#### Initialization

The position of the lions is kept in a matrix, and the population is produced at random throughout the solution space. The population over the solution space is randomly generated as the initial stage in LOA. Every solution in LOA is referred to as a lion, which is symbolized by.

2 $$Lion=[{x}_{1},{x}_{2},\dots{x}_{{N}_{var}}$$

Where ${x}_{{N}_{var}}$stands for nomad lion ratio in this study is %N produced at random, while the balance of the population is made up of resident lions. Here, the number of top CH picks is taken into consideration as the input solution.

By looking for and identifying undiscovered links between persons in a particular network, LSOA is used to enhance CH selection.

**Fitness calculation process**

Each lion’s fitness value is determined by analyzing the objective function before being sorted and recorded in a matrix.

3 $$f\left(Lion\right)=f({x}_{1},{x}_{2},\dots{x}_{{N}_{var}})$$

Fitness calculations take into account delays and energy, thus the best partition for successfully transmitting an emergency message is selected as CHs. The settings for the fitness function employ delay and minimal energy usage.

4 $$Fitness=Min\frac{\sum_{i=1}^{N}({P}_{energy\, consumption}^{i}+{P}_{delay}^{i})}{2}$$

Here P^i^_energy consumption_ displays each node’s energy use after getting the data from the chosen CHs. In the selection nodes, the P delay i takes place. In Eq. (4) and, the formula for delay and energy usage is provided (5). The process of updating the answer will be completed after the fitness calculation. The main updating processes in the lion algorithm are defence, roving, mating, and hunting. In the pride’s territory, few female lions hunt for prey, and some will settle in the same area. Each region has its own top qualities, which will aid in determining the best on each iteration. Based on fitness, CHs were selected from initial solutions while the best among them were chosen from updated solutions. Several performance criteria, such as latency, malware detection accuracy, coverage, and energy efficiency, are assessed and optimized using the fitness function. Energy efficiency is defined as the ratio of energy saved to energy consumed; coverage is defined as the area under management or the percentage of nodes that have been successfully handled; malware detection accuracy is defined as the ratio of threats that have been accurately identified; and latency is defined as the time it takes for a system to respond. These metrics are weighted together to form this metric. To make them all comparable, we normalize them and then weight them all together to get a single fitness score that this study uses to optimize our optimization. This method guarantees that the LSO and ECC scheme successfully balances these elements to enhance network performance and security by meticulously adjusting the weights and evaluating the function using simulations or real-world tests.

**Hunting**

Three groups are randomly selected from among the hunters. The hunter with the best physical condition is selected as the center, leaving the left and right arms as the two surviving hunters. At the moment the fake prey is attacked, one hunter is randomly selected. If the hunter becomes more fit and the new location is updated, the prey flees.

**Moving toward safe place**

In each pride, only a small number of female lions focus on hunting. The rest of the women moved to a safer area of the property. The ideal posture for each pride is calculated and maintained. A large number of victories indicate that the lions’ combination is still far from being at its best. Additionally, a lack of successes illustrate that the lions are circling the optimal configuration lacking of major enhancement. As a result, the achievement esteems are used to evaluate the competition’s measure.

**Roaming**

Roaming is one of the difficult and confined search strategies employed by LSOA in order to investigate the search space and enhance the result. The lion goes n units in the direction of its preferred territory where n is a uniformly distributed random integer.

5 $$n\sim U\left({0,2}*d\right)$$

Where d is the distance among the male lion’s location and the preferred area. Also, the nomadic lion roams aimlessly throughout the search area.

**Mating**

The crucial process that ensures the lions’ survival is mating. It involves producing two new children from the parents. The right male and female lions are chosen, and then the cubs are born. Crossover and mutation are two processes that provide people the chance to create new, better solutions from those already available. Killing unhealthy or weak cubs makes sure that the solutions that result are the best.

**Defence**

Defence is one of the most crucial behaviours in a lion’s everyday existence. The adult male lions in that pride become aggressive, destructive, and engaged in combat with other lions. Male lions that have been defeated are expelled from the pride or go on the move. If a nomad lion feels physically fit and challenges the other lions in the pride, but fails, it will then join the pride as a resident lion and drive the current resident lion out. The user must follow two steps.

- Protection from newly arrived mature resident men.
- Protection against male nomads.

The strongest and most powerful lion in the pride is identified by this operator.

**Migration**

For this migration process, a random selection of female lions is made, and they become nomads. The highest fitness values of the new and elderly nomads determine where they are placed. To fill the role of moved or migratory female lions, the female lions in the pride with the greatest fitness level are replaced.

**Lions’ population equilibrium**

**Fig. 3:**
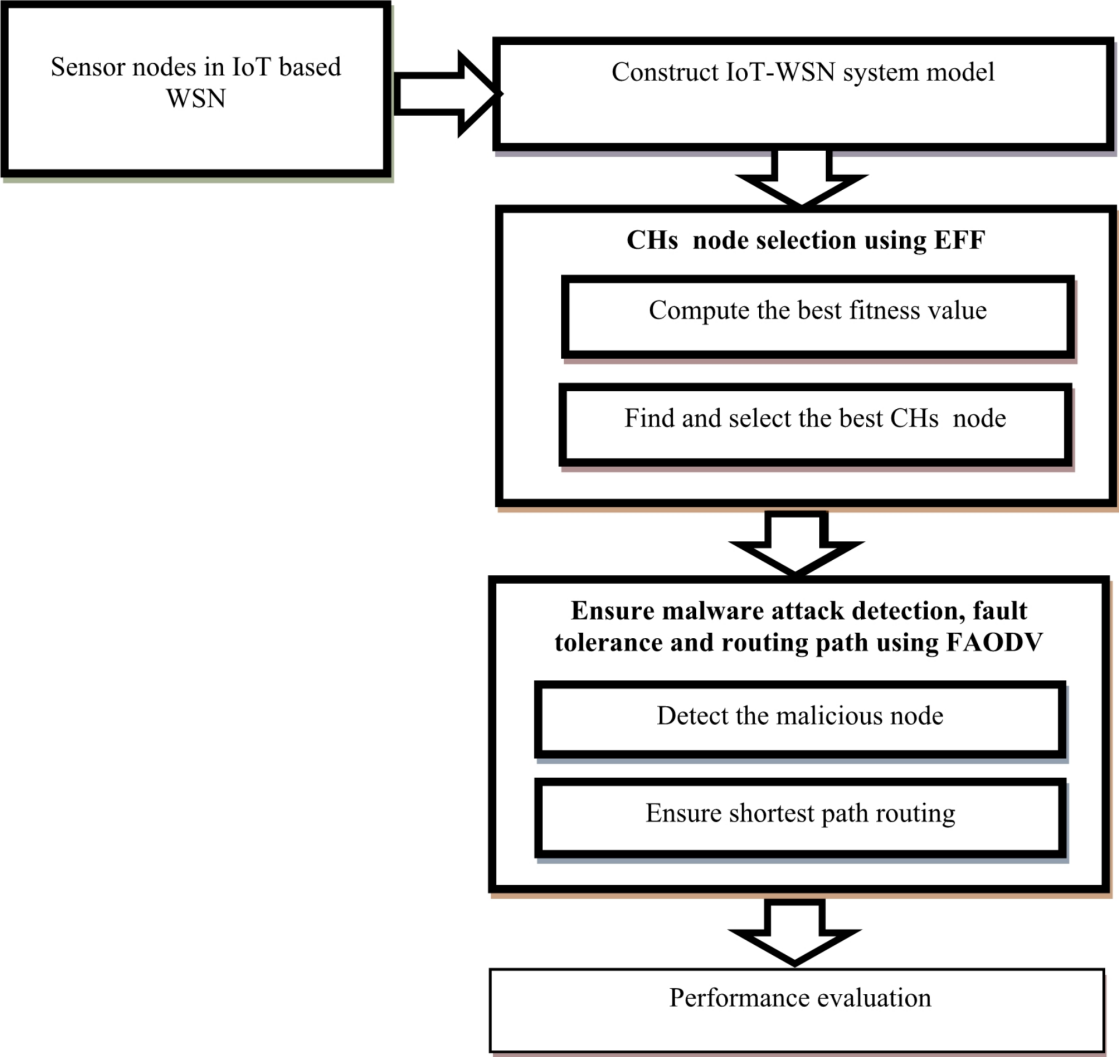
Block diagram of present ELSO with ECC framework.

The lion population maintains a constant point, or equilibrium, at the conclusion of each cycle. There is a cap on how many lions of each gender are allowed in the nomad category. The population eliminates the lion with the lowest degree of fitness. The shortest distance between nodes is represented in Fig. 3. It reduces cluster size closer to the base station and raises cluster size as the distance among the cluster head and the base station rises. In our approach, the routing algorithm for shortest path selection is based on a combination of Lion Swarm Optimization (LSO) and a standard shortest path algorithm. The primary goal of the routing mechanism is to ensure efficient data transmission while minimizing energy consumption and network delays.

**Termination criteria**

This method terminates the process if a significant number of iterations are finished with the best fitness. In this situation, the division with the highest fitness is picked as the one in which the message should be broadcast first. The compete for territory between the two territorial males is evident. In doing so, the algorithm will preserving the better solutions while removing the undesirable ones (section operation) only storing the best male and female solutions over fresh responses and deleting any other solutions.

The ELSO method, which is based on ant location updates on random walks around the ant-lion optimizer chosen by the Roulette wheel and the elite, retains the best particle by including the elite into the search process. These factors contribute to ELSO has quick calculation times, greater efficiency, and firm convergence^18^. Based on how ant lions hunt, an optimization algorithm was created. This algorithm uses a random walk for agent exploration and random selection. Traps are used in the exploitation process. ALO encouraged us to apply a placement strategy for Dynamic Generators (DGs) that, to the authors knowledge, had never been employed before. Ant Lion Optimizer (ALO) is recognized for its remarkable capacity to resolve complex optimization issues. To efficiently explore huge solution areas and discover optimum solutions, it emulates the hunting behaviour of antlions. Tasks like cluster head selection in IoT and WSNs, which require balancing many aspects, including energy efficiency, network coverage, and resource limits, are especially well-suited to ALO. System performance optimization and configurations in dynamic contexts are made possible by the algorithm’s capacity to overcome these obstacles effectively and converge to near-optimal solutions.

It primarily uses five hunting techniques: a random walk of agents, the construction traps, the entrapment of ants in trap, catching prey and rebuilding traps. Local optima can be eliminated via the ALO optimizes roulette wheel and ant-walk randomization. The phenomena of early convergence and local optimum appear for complex optimization problems. Certain improvements are included in this CHs selection technique to increase optimization capability and lifespan.

6 $$Ant=\frac{{R}_{A}+{R}_{E}}{2}$$

Where Ant is the new location, R_A_ is the ant-lion walk around picked at random, and R_E_ ant-lion walk around chosen at random for the elite. If the ant’s new position exceeds the beyond, changing it.

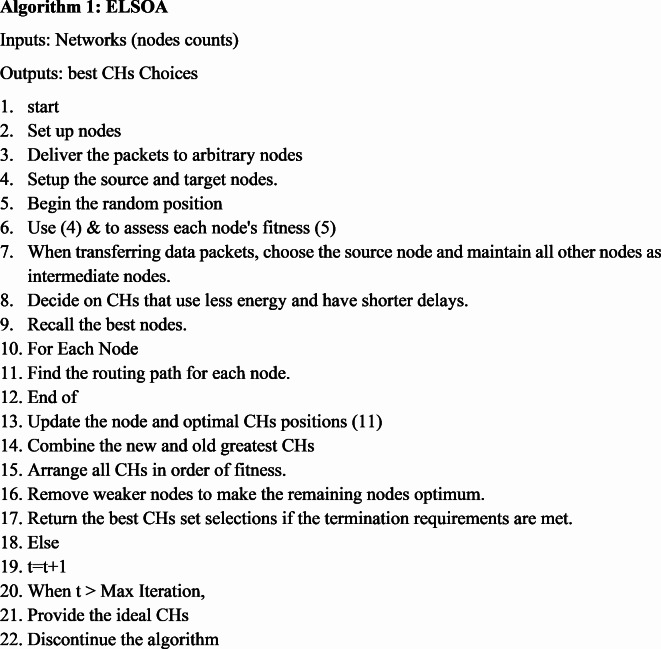



This method explains the stages taken from the lion optimization algorithm. 1. Beginning with a randomly selected population of potential solutions (lions), the algorithm proceeds to formulate a model. ELSO is a method for discovering solutions by simulating lion activities like hunting and interacting with others. The lions’ placements are adjusted using their fitness values and previously established rules in each cycle. As the algorithm iterates, it considers the lions’ fitness and the best solutions discovered so far to adjust their placements. ELSO stops when the specified number of iterations or convergence conditions are satisfied. The speed at which objects come together depends on the hybrid strategies and upgrades. Lions are the world’s strongest mammals because of their distinctive social behaviour. Lions have two distinct social behaviour patterns: residents and nomads. Residents may transition to nomadic behaviour and vice versa. Male and female residents give birth in pride groups living communities. Nomads, the second organizational behaviour, are sporadic movers who may travel alone or in pairs. Related guys left out of their mothers’ pride tend to form pairs more frequently.

### Optimal path routing and attack detection using MHD and ECC scheme over IoT based WSN

This study uses the MHD approach to perform optimum path routing. According to where each node in the network is located, BSs gathers information about all of its neighbour. In order to create the hop database, we first split the nodes into N sections based on the distance among the regular node and the sink node as well as the relationships among neighbour. To tell all network nodes on the hop information, BSs will broadcast it. Median Bit Difference (MHD), s a statistic used to compare two data sequences and find the number of bits that vary between each. Utilizing MHD, incoming data packets are compared to recognized lawful patterns within the context of attack detection. A data injection or manipulation assault, which might affect the system’s dependability, can be indicated by a significant variation in the Hamming distance. MHD looks for unusual discrepancies in data patterns to assist in finding unlawful changes in real time. In contrast, Elliptic Curve Cryptography (ECC) is used to encrypt data transfer securely, even while using lower key sizes than standard cryptographic systems. Protecting data transmitted between nodes from unwanted access and alteration is key to ECC. Because ECC-based cryptographic signatures are not identical during verification, an attacker trying to disrupt the transmission will be identified.

It establishes M routes to the Sink during the shortest hop distances after randomly adding M nodes to the source node collection. It transfers the data to the relay node until it reaches the Sink, recording the node ID in the routes. To every route set, we append the node ID${C}_{i}=(i={1,2},3,\dots M)$ for node j, if

7 $$J=\left\{k\right|\underset{k\in neighbor\left(i\right)}{{\min}}hop\left(k\right)$$

When the route packets reach the Sink, node J adds to set Ci in accordance with the aforementioned principles. The Sink determines the frequency of M routes passing through one node, or the happening numbers of node i. were

8 $$C={\cup}_{i=1}^{M}{C}_{i}$$

Take Record of the M routes through each disjoint node. If $count\left(x\right)>\alpha$

The node is malicious node, if $\beta\le count\left(x\right)\le\alpha$meets the formula

The hop needs to be tested further. The detection rate is impacted whether M is large or small in areas where a; b is rising functions around M.

However, if M is too large, the energy consumption is higher and the false positive rate is lower. In order to balance network performance, it must choose the proper value.

9 $$\left\langle {hop} \right\rangle=\frac{\sum_{i=1}^{m}hop\left(i\right)}{m}$$

10 $$\Delta\left(x\right)=\frac{\left\langle {hop} \right\rangle-hop\left(x\right)}{\left\langle {hop} \right\rangle}$$

Where m is node x’s number of neighbour. If $\Delta\left(x\right)$ is exceeds than the threshold d, and the node x is found to be malicious node.

**Secured data transmissions using ECC technique**

ECC is used in this study to improve attack detection and security when transferring data through WSN. Every user or device participating in the communication typically possesses a pair of keys—a public key and a private key—as well as a set of actions associated with the keys in order to perform cryptographic operations. This is known as public key cryptography. While only one user has access to the private key, everyone else involved in the communication has access to the public key. It is used to generate faster, smaller, and more effective cryptographic keys.

Routing attacks of the type called sinkhole attacks always include at least one rogue node. Typically, it announces to the target node that it has a superior data send path. The attack’s malicious node, which has the capacity to alter the network’s routing protocol, is often placed close to the Sink node. It results in data packets being transferred to malicious nodes when they should have been delivered to the sink node. Since all of the packets in WSN have the same destination due to their unique transmission mode, the malicious nodes just need to establish a reliable link with BS.

ECC, which offers higher security, is implemented to solve the aforementioned issue. The sender encrypts the communication utilizing the recipient’s public key in the ECC algorithm and the recipient’s private key is used to decode it^20^. The number ’d’ must now be selected from the available range of ‘n’. With the help of the subsequent equation, it may produce the public key.

11 $$Q=d*p$$

Where, d = The number of random that we have choose within the range of (1 to n-1).

P = the point on the curve. Q = the public key and d is the private key.

Although proposed approach only used ECC encryption for IDs, all incoming sensed data from sensor nodes are encrypted using ECC. As a result, the work demonstrated higher energy consumption outcomes. In this investigation, unlike CHs status response messages and data messages, only one message from sensor nodes with all essential data for verification were transmitted to CHs. The simulation results demonstrated that this method completely secured networks in implementations.

Figure 3 shows the Block diagram of present ELSO + ECC framework. Nodes transmit messages to sensor nodes with encrypted IDs if they start act as sinkhole attackers. The encrypted IDs of the sender nodes are sent to CHs for authentication when other nodes receive this message. If CHs determine that these IDs belong to sensor nodes rather than CHs, or if IDs disclose that these nodes are a part of clusters at lower levels, nodes are assumed to be attackers. CHs then swiftly reports the attack to BSs in an alarm message, along with the attacker’s ID, and sends NACK (Negative Acknowledgement message) to the receiving node, preventing contact with the attack node.

In the exploration phase, α controls how much the algorithm explores the solution space. Stricter investigation is encouraged by a lower α value, while a more significant value limits the search to more promising places. Contrarily, β dictates how the algorithm improves upon the present best answers and impacts the exploitation phase. While a lower β encourages more investigation, a higher β focuses on using the optimal solutions. The values of α and β are usually found by experimenting, adjusting the parameters, or performing sensitivity analysis, which ensures that the optimization process finds an appropriate balance between exploring and exploiting that matches the circumstances.

As a result, there will be significantly greater privacy because the attacker won’t be able to eavesdrop on the data from intermediary nodes. It offers security from a various attack, including changing the routing information sinkhole, black hole, wormhole, and Sybil attacks, among others. Additionally, it employs encryption as a security feature to safeguard messages. In order to provide safe data routing from sensor nodes to BSs, the recommended structure is used.

In a clustering-based approach, nodes are arranged into groups known as clusters, with a designated leader for each cluster. The cluster head is responsible for data aggregation and transmission to the base station or sink. The transmission distance is drastically decreased by storing data at the cluster head instead of each node communicating with the base station individually. Shorter transmissions need less energy hence this decrease in distance decreases the data transmission energy requirement. Cluster nodes reduce communication range and energy costs by communicating with the cluster head instead of transmitting data across vast distances to the base station.

## Simulation result

In this part, the current ELSO + ECC strategy is evaluated and compared to other methods like FedGANs. The present approach and the recommended way are compared in terms of end-to-end latency, throughput, energy use, packet delivery ratio, and network durability. MATLAB was chosen because it offers comprehensive tools for simulating IoT and WSN systems. These tools support the design of cluster-based architectures and allow for effective evaluation of the Lion Swarm Optimization (LSO) algorithm and Elliptic Curve Cryptography (ECC). This study analysed the proposed method in networks with up to 500 nodes to demonstrate its scalability and robustness across larger IoT-WSN environments, which are increasingly common in practical applications. For nodes below 100, while the performance metrics show a notable improvement, this study opted for the higher range to emphasize the stability and efficiency of the approach under more extensive network conditions. Our study selected the source and destination nodes based on typical IoT-WSN deployment scenarios, where data is collected from sensor nodes (sources) and transmitted to a central base station or designated node (destination).

ELSO improves the exploration-exploitation equilibrium by including upgraded mechanisms like adaptive management strategies and dynamic updating of search space limits. Because of this, the method can escape local optima more successfully than classic Lion Optimization Algorithms (LOA). Applications may modify ELSO’s fitness assessment criteria to meet their goals, such as optimizing cluster head selection in IoT networks or reducing energy consumption, resulting in optimization results that align with the intended use. For a more reliable search over complicated and high-dimensional solution spaces, ELSO uses population diversity management and sophisticated initialization approaches. Finally, ELSO shows theoretically improved performance in metrics like latency, energy efficiency, and network lifetime thanks to its hybrid integration with domain-specific restrictions and problem-tailored heuristics.

The simulation parameters appropriately represented real-world circumstances in IoT and WSNs to ensure the findings were relevant to real-world settings. The selected parameters, like node density, network size, and mobility patterns characteristic of Internet of Things (IoT) applications, allowed for a thorough assessment of the ELSO algorithm’s performance and scalability in different network topologies. The selection of latency, energy efficiency, and network lifespan as performance indicators is based on their ability to tackle essential facets of IoT network performance. Applications that need low-latency communication place a premium on delay. In contrast, WSNs place a premium on energy economy due to optimizing battery-powered nodes needed to preserve resources. A vital indicator of a network’s stability and efficacy over time, network longevity measures the algorithm’s influence on its short-term performance and capacity to run efficiently in the future (Table 2).

**Table 2:** Simulation parameters.

Parameter	Values
Nodes	100 to 500
Size of the Area	1000 * 1000(Meter)
Mac	802.11
Overall energy	100 J
Starting energy	1.0 J
Radio Range	250 m
Simulation Time	200 s
Size of the Packet	512

The average time it takes for a packet to move from its source to its destination across a network is referred to as end-to-end delays.

12 $$End-to-enddelay=\frac{\sum_{i=1}^{n}({t}_{ri}-{t}_{si})}{n}$$

Where t_ri_ is the accept time of i^th^ packet, t_si_ is the sending time of i^th^ time and n is the overall amount of packets.

**Fig. 4:**
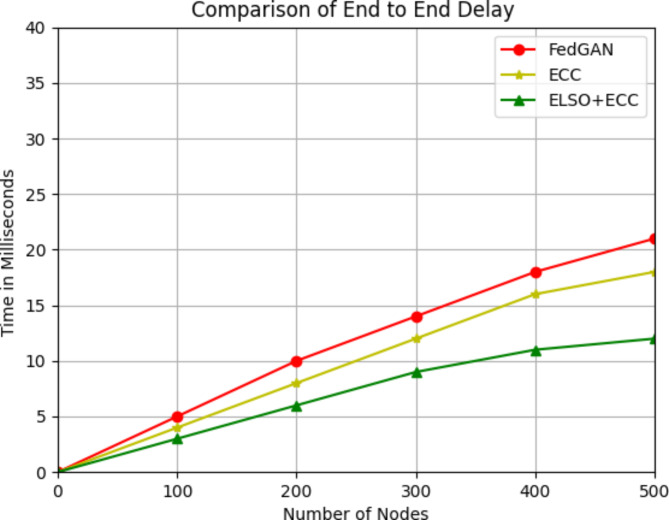
End-to-end delay comparison with existing methods.

**Fig. 5:**
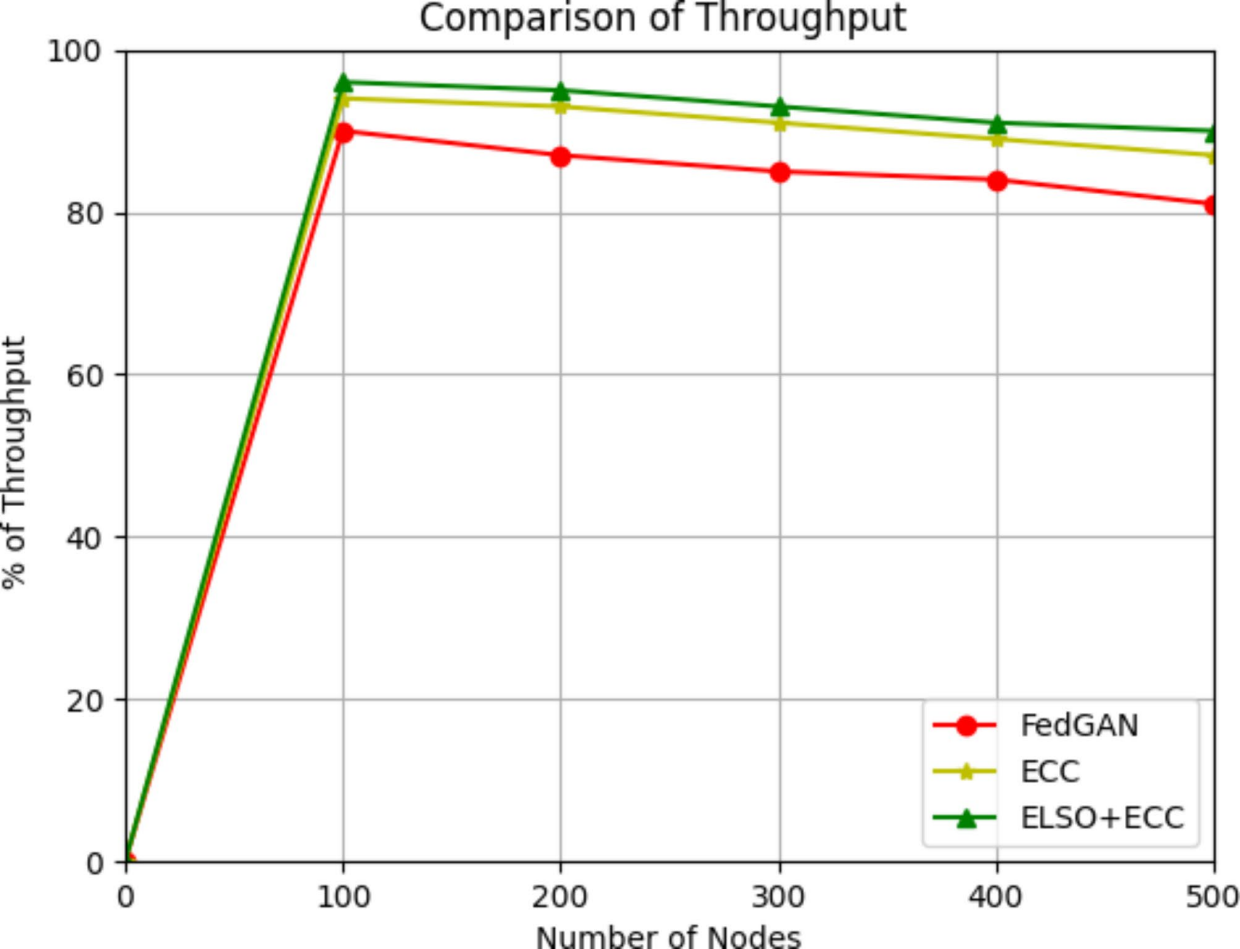
Throughput comparison with existing methods.

Figure 5 comprises the end-to-end delay act of the present FedGAN approach, the ECC algorithm, and the present ELSO + ECC algorithm. The numerous of nodes is shown by x-axis and the end-to-end latency is depicted by the y- axis. The present ELSO approach for IoT-based WSN significantly decreases the time required to deliver data during the secured data transmission process. CHs use a number of ways to get the necessary data from the higher energy sensor nodes. In IoT-based WSNs, the suggested ELSO + ECC algorithm offers effective path routing for detecting attacks. In round 1 out of 100 nodes the end-to-end delay was 0.3 milliseconds in ELSO + ECC where as in 0.4 in ECC and 0.5 in FedGAN likewise for 500 nodes in proposed ELSO + ECC the End-to-End delay is 12 milliseconds where as other existing protocols are 18, 21 milliseconds. It shows in each round ELSO + ECC makes a significant performance in the End-to-End delay of the nodes are decreased. In Fig. 4, the green bar for the combined approach does appear higher across different node counts and considers the context in which this energy usage is measured. The combined approach, which integrates Lion Swarm Optimization and Elliptic Curve Cryptography (ECC), includes additional processing for security enhancements that inherently increase energy consumption compared to non-secure or less optimized methods.

Throughput is the rate at which the data packets are effectively sent via a network or other communication channels.

13 $$Throughput=totalEquationNumberofpacketssent/time$$

For the throughput metric, Fig. 5 compares the existing FedGAN, ECC, and new ELSO + ECC algorithms. The number of nodes is measured on the x-axis, while the throughput metric is measured on the y-axis. In IoT-based WSN, the suggested ELSO + ECC algorithm is utilized to successfully identify attacks utilizing route discovery and maintenance criteria. This facilitates precise data collection and transmission over several nodes without data loss. In round 1 out of 100 nodes the throughput was 96% in ELSO + ECC where as in 94% in ECC and 90% in FedGAN likewise for 500 nodes in proposed ELSO + ECC the throughput is 90% where as other existing protocols are 87% and 81%. It demonstrates that the recommended ELSO with ECC technique has a greater throughput than the existing FedGAN, ECC methods. As the number of nodes in the network grows, throughput often decreases for various reasons. Network congestion results from more data traffic, which grows as the number of nodes increases. With more packet collisions and retransmissions caused by congestion, bandwidth consumption rises, and effective throughput falls. Secondly, in an environment where several nodes are vying for a single communication channel, the throughput is further constrained since each node takes longer to transfer data successfully. At last, in IoT-WSN situations, more nodes mean more energy consumption per node as they try to communicate more often, which means nodes may run out of extract quickly, limiting their data transmission capabilities. When taken as a whole, this causes the throughput to drop as node density rises.

Energy consumption are averages of energy necessary for transmissions while receiving or forwarding packets to nodes in networks during a period of time

14 $$Energy\left(e\right)=[\left(2*pi-1\right)\left({e}_{t}+{e}_{r}\right)d$$

Where pi is the data packet, e_t_ is the energy for transmitting packet i, e_r_ is the energy for receiving packet i and d is the distance among the transmission node and destination node.

**Fig. 6:**
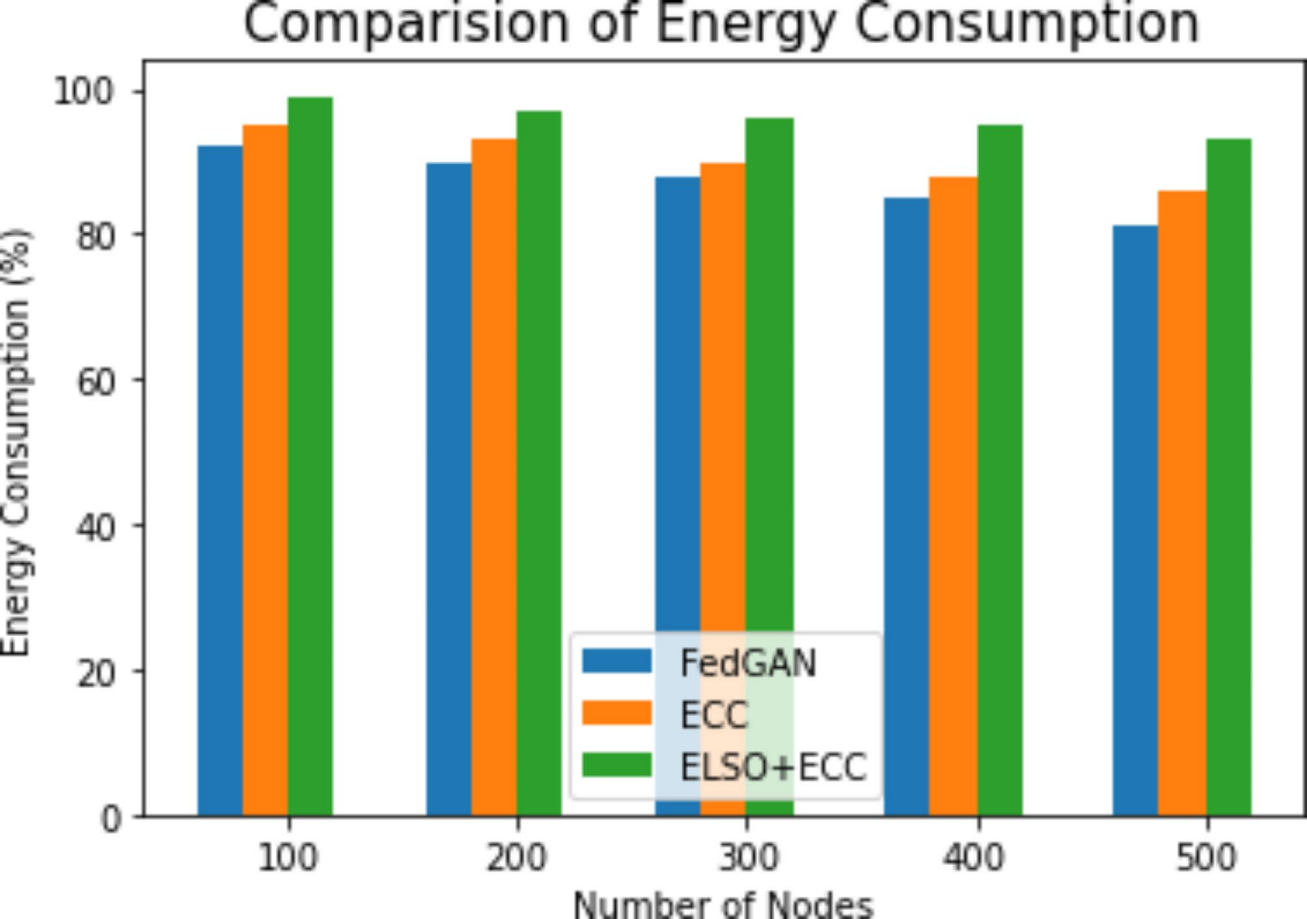
Energy consumption comparison with existing methods.

It is evident from Fig. 6 that the present ELSO + ECC algorithm and the current FedGAN, ECC, and FedGAN algorithms were measure for energy usage. The x-axis measures the number of nodes, while the y-axis measures the energy consumption metre. The suggested MHD algorithm over the IoT based WSNs greatly decrease usage of energy for the period of the data packet transmission. The use of CHs-based data allows for the classification of sensor nodes according to their degree of residual energy. The energy usage node, which is intended to construct optimum path routing, is improved using ELSO. It demonstrates that the ELSO + ECC algorithm, which has been developed, uses less energy than the currently used approaches.

When the recommended solution offers a lengthier network lifetime, the system is performed better.

15 $$Lifetime\mathbb{E}\left[L\right]=\frac{{\epsilon}_{0}-\mathbb{E}\left[{E}_{w}\right]}{P+\lambda\mathbb{E}\left[{E}_{r}\right]}$$

Where P is the persistent continuous power consumption of the overall network, ${\epsilon}_{0}$ is the overall non-rechargeable initial energy, $\lambda$ is the average sensor reporting rate, $\mathbb{E}[{E}_{w}$] is the predictable wasted energy or unexploited energy when the network dies and $\mathbb{E}\left[{E}_{r}\right]$ is the predictable reporting energy spent by all sensors

**Fig. 7:**
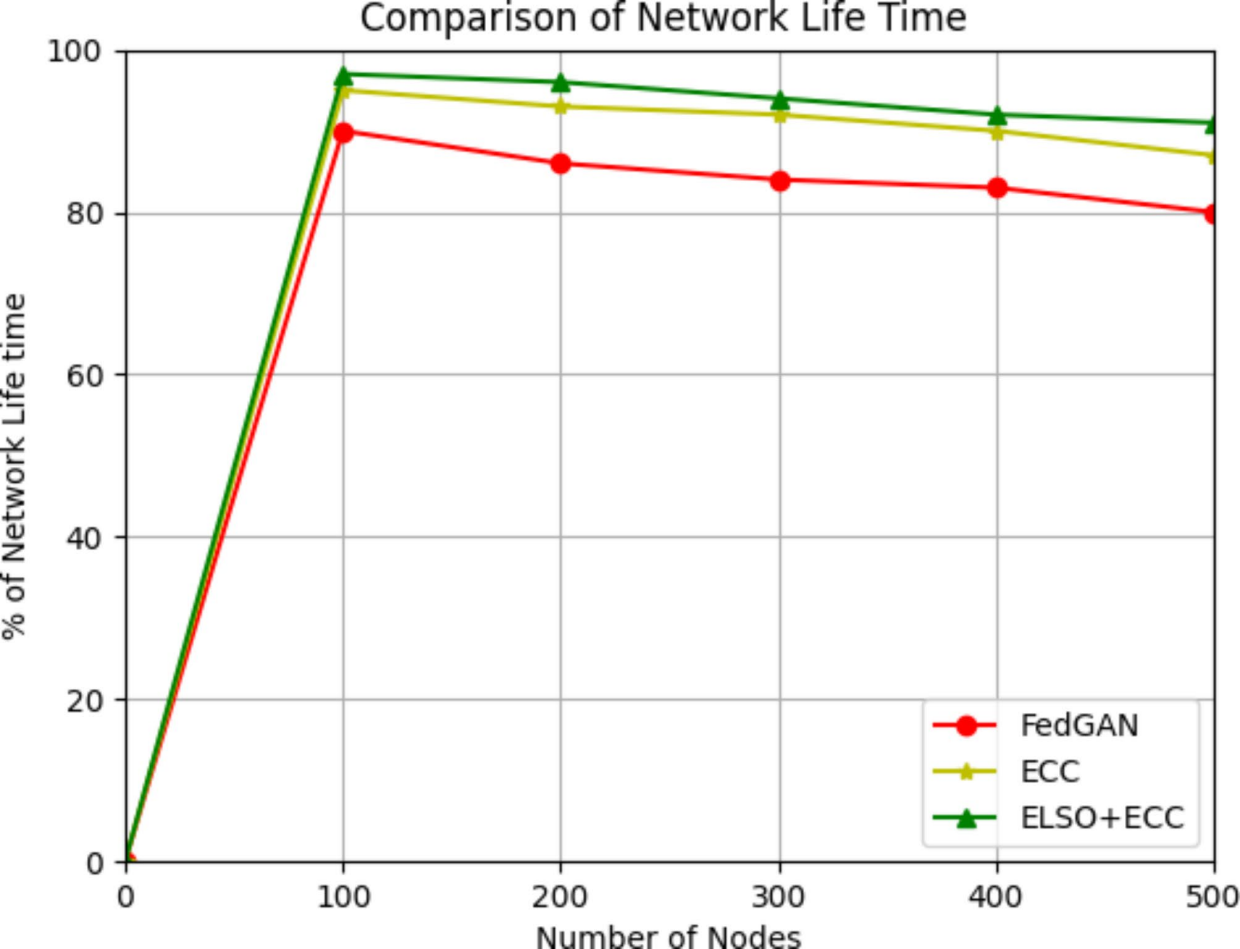
Network lifetime with existing methods.

The network lifespan for the specified size of the packet is shown in Fig. 7. Quantity of nodes is measured on the x-axis, while network life cycle is measured on the y-axis. The suggested ELSO + ECC method considerably increases the lifespan of the sensor node during data packet transmission. This is as a result of employing the ELSO algorithm to accomplish CHs-based data transfer. By applying the ECC method to prevent attacks, a large number of packets may be transferred. Additionally, it has been noted that the multi path routing employed by the proposed system lengthens the time complexity. In round 1 out of 100 nodes the life time of the network is 97% in ELSO + ECC where as in 95% in ECC and 90% in FedGAN likewise for 500 nodes in proposed ELSO + ECC the life time of the network is 91% where as other existing protocols are 87% and 80%. It demonstrates that the suggested ELSO + ECC algorithm offers a longer lifetime of the network than the other FedGAN and ECC methods now in use. The ELSO algorithm and ECC scheme greatly enhance network performance, security, and efficiency by reducing computational complexity. Costs in computing, communication, and energy are all part of the overheads related to these enhancements. To ensure that the network’s advantages exceed the costs, the study optimizes algorithm environments, uses efficient ECC implementations, and uses adaptive methodologies to handle and control these overheads. This method can build a more secure and efficient IoT network while keeping operating costs low.

PDRs are defined as the sum of packets effectively received by the destination. Since nodes are usually placed close together to maximize communication and minimize energy consumption, the tiny area chosen for this research reflects typical IoT-WSN deployment conditions. Nodes in applications like smart buildings, precision agriculture, and industrial monitoring often cover small areas, collect data from surrounding nodes, and send it to a base station or gateway. Decreased transmission energy allows us to shorten the distance between nodes, increasing the network’s longevity and dependability. This study opted for data packets in the kilobyte or byte range to represent the small data packets often seen in IoT applications, including sensor readings or status updates. Smaller packets save transmission time and lower the chance of packet loss in networks with limited capacity, making them well-suited for energy-constrained IoT nodes. With this method, we may meet the data transmission requirements of IoT-WSN applications, which value small, frequent transfers of data above large, rare ones and aim to save energy.

**Fig. 8:**
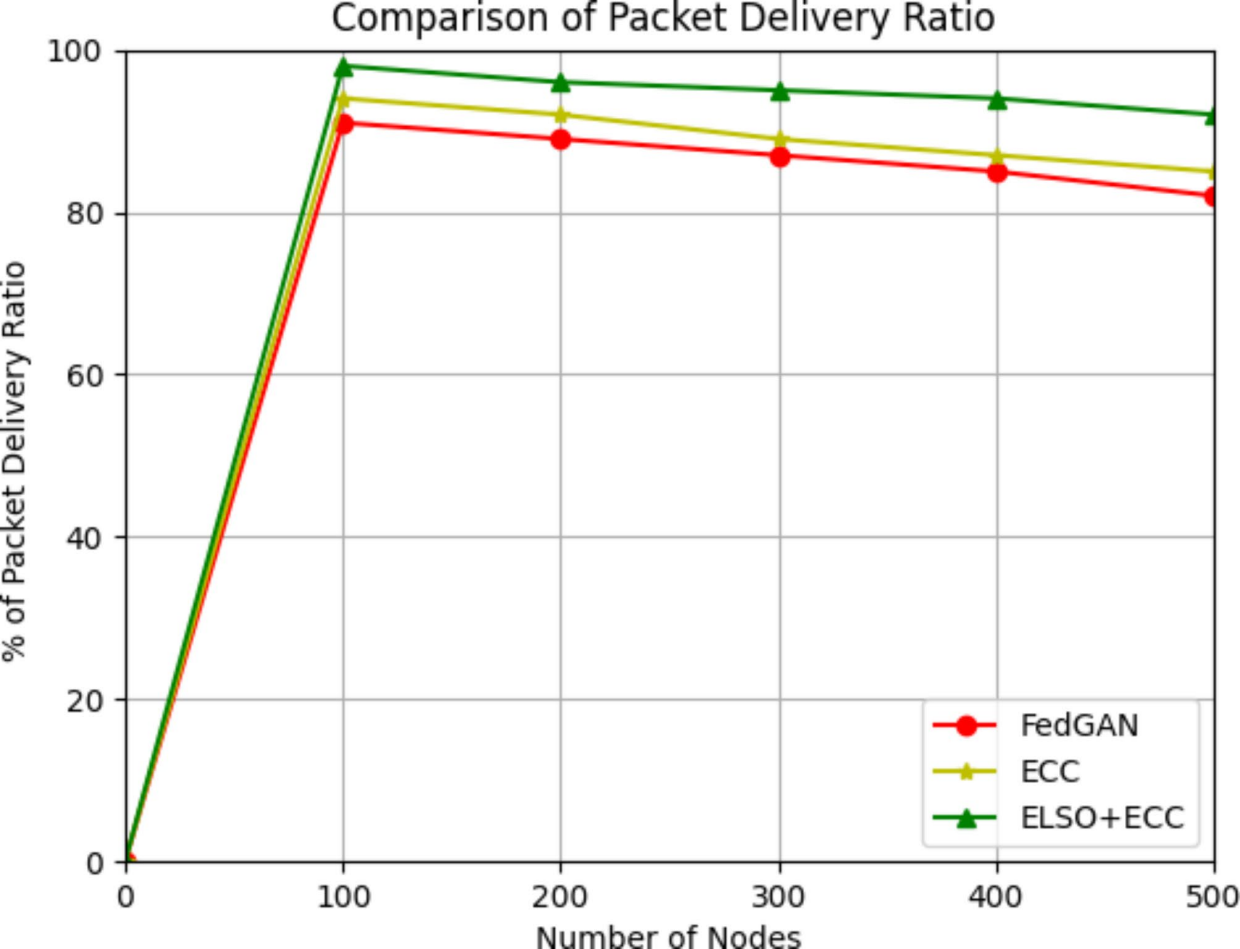
Packet delivery ratio with existing methods.

The new ELSO + ECC algorithm and the existing FedGAN approach are evaluate in terms of packet delivery ratio in Fig. 8. The quantity of nodes is depicted on the x axis, while the PDR values are depicted on the y axis. The PDR values in the current scenario are decreased using FedGAN and ECC approaches. The PDR value in the proposed system is considerably increased by using the provided ELSO + ECC approach. In round 1 out of 100 nodes the packet delivery ratio is 98% in ELSO + ECC where as in 94% in ECC and 91% in FedGAN likewise for 500 nodes in proposed ELSO + ECC the time complexity is 92% where as other existing protocols are 85% and 82%.

**Fig. 9:**
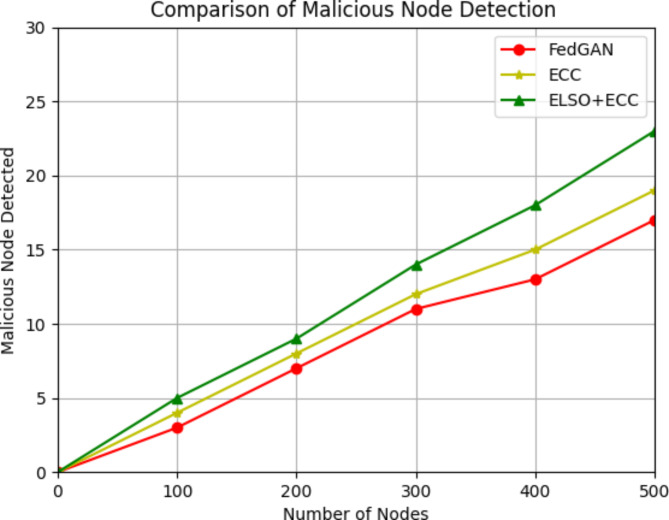
Comparison of Malicious node detection with existing methods.

The above figure shows that malicious node detection rate as small compared with the existing approaches. Consider 5 malicious nodes for 100 nodes in all out of 500 nodes with the malicious nodes as 25. The ELSO + ECC d method can detect out of 25 nodes 23 nodes are indicating the efficiency of proposed method compared to other previous protocols. It is shown in the Fig. 9. Even though numerous of nodes deployed in every round are increased, the malicious activity is controlled by prevention. As a result, it illustrates how the proposed ELSO + ECC approach is employed to achieve safe and well-organized data transmission in WSNs.

Integrating adaptive methods that enhance solution exploration, ELSO increases convergence speed and decreases the time needed to get optimum results. Conventional methods, such as the Lion Optimization Algorithm (LOA), may get stuck in local optima; this technique achieves a better balance among exploration and exploitation. Additionally, the algorithm has better energy consumption optimization, meaning that battery-powered sensor nodes may stay operational longer, and it scales efficiently with the complexity and size of extensive IoT networks. The fact that ELSO may be adjusted according to specific performance measures like latency, energy economy, and network lifetime further adds to its adaptability. Compared to other methods, ELSO is more flexible and efficient for real-world applications because of its resilience in dynamic contexts, where factors such as node failure or mobility are customized.

It is challenging to apply the “ELSO-ECC” technique in practical Internet of Things (IoT) deployments because IoT devices frequently have limited resources, such as processing power, memory, and battery life. Although ECC is not as heavy as other cryptographic techniques, it can increase the computational and energy needs. This might place a burden on devices and affect the lifetime of the network. In addition, low-latency replies are crucial in large-scale networks, and Lion Swarm Optimization’s repeated communication between nodes for clustering might cause latency concerns. Environmental dynamics in IoT environments might compromise malware detection and secure communication, including interference, device mobility, and fluctuating signal intensities, which could affect the algorithm’s stability and efficiency. While ECC does improve security, it can still need additional compliance requirements for sectors with strict data standards, which might make adoption more difficult. Not all Internet of Things (IoT) devices have the necessary cryptography and optimization capabilities, which further complicates matters and highlights the need for specialized adaptations in heterogeneous networks.

## Conclusion

The ELSO integrated with ECC strategy has been suggested and employed in this study for optimizing the choice of CHs, securing data transmission, and attack detection via IoT with WSNs. The ELSO algorithm has been recommended as a method for the identification of the optimum CH choices. The finest fitness values have been utilized the selection of CHs. When selecting between CHs, the best outcome is gained by taking into account the fitness function of the remaining energy and the end-to-end delays. MHD is used in route routing for a decrease in the number of hop count nodes. Next, data transmission is secured using the ECC approach for enhancement of the functionality of WSNs. Elimination of sinkhole attacks and black hole nodes, helps getting a significant reduction in packet loss. The suggested ELSO with ECC solution outperforms the substitutes in terms of the throughput, network durability, PDR, end-to-end latency, and energy usage. The major emphasis of the future study will be the creation of new routing algorithms for transferring data from the source to the sink. This strategy will use meta-heuristic optimization approaches to address the challenges of topology building, data routing, and loss tolerance. Enhancing the system’s scalability and flexibility for controlling large-scale IoT networks with variable node density and dynamic situations might be the subject of future study. It can improve accuracy and real-time responsiveness by incorporating powerful machine-learning algorithms into virus detection. Lightweight cryptographic techniques may improve energy usage while maintaining solid security measures. Data integrity and resistance against possible assaults may be enhanced by incorporating blockchain technology for decentralized data validation and security in wireless sensor networks.

## Data Availability

The datasets generated during and/or analysed during the current study are available from the corresponding author on reasonable request.
